# Monitoring Growth and Removal of *Pseudomonas* Biofilms on Cellulose-Based Fabrics

**DOI:** 10.3390/microorganisms11040892

**Published:** 2023-03-30

**Authors:** María del Rosario Agustín, Peter Stengel, Matthias Kellermeier, Katrin-Stephanie Tücking, Mareike Müller

**Affiliations:** 1 Instituto de Ciencias Biológicas y Biomédicas del Sur, INBIOSUR (UNS-CONICET), San Juan 670, Bahía Blanca 8000, Argentina; mariadelrosarioagustin@gmail.com; 2 Departamento de Biología, Bioquímica y Farmacia, Universidad Nacional del Sur (UNS), Bahía Blanca 8000, Argentina; 3 BASF SE, Carl-Bosch-Str. 38, 67056 Ludwigshafen, Germany; peter.stengel@basf.com (P.S.); matthias.kellermeier@basf.com (M.K.); katrin-stephanie.tuecking@basf.com (K.-S.T.); 4 Physical Chemistry I & Research Center of Micro and Nanochemistry and (Bio)Technologie (Cμ), Department of Chemistry and Biology, University of Siegen, Adolf-Reichwein-Straße 2, 57076 Siegen, Germany

**Keywords:** biofilm removal, *Pseudomonas* spp., cellulose, textiles, enzyme, detergent

## Abstract

Biofilms are often tolerant towards routine cleaning and disinfection processes. As they can grow on fabrics in household or healthcare settings, resulting in odors and serious health problems, it is necessary to contain biofilms through eradication strategies. The current study proposes a novel test model for the growth and removal of biofilms on textiles with *Pseudomonas fluorescens* and the opportunistic nosocomial pathogen *Pseudomonas aeruginosa* as model organisms. To assess the biofilm removal on fabrics, (1) a detergent-based, (2) enzyme-based, and (3) combined formulation of both detergent and enzymes (F1/2) were applied. Biofilms were analyzed microscopically (FE-SEM, SEM, 3D laser scanning- and epifluorescence microscopy), via a quartz crystal microbalance with mass dissipation monitoring (QCM-D) as well as plate counting of colonies. This study indicated that *Pseudomonas* spp. form robust biofilms on woven cellulose that can be efficiently removed via F1/2, proven by a significant reduction (*p* < 0.001) of viable bacteria in biofilms. Moreover, microscopic analysis indicated a disruption and almost complete removal of the biofilms after F1/2 treatment. QCM-D measurements further confirmed a maximal mass dissipation change after applying F1/2. The combination strategy applying both enzymes and detergent is a promising antibiofilm approach to remove bacteria from fabrics.

## 1. Introduction

Textiles, particularly those composed of natural fibrous materials, such as cotton, linen, or wool, are widely used in healthcare, institutional, and household settings and, like other polymeric materials, are susceptible to contamination by various microorganisms deriving from the environment and human skin, including pathogenic bacteria, viruses, yeasts, and spores [1,2]. The presence of organic materials in textiles offers an excellent substrate for microbial proliferation, since they provide a good base for attachment of microorganisms. Furthermore, human sweat, which is retained by textiles, provides nutrients necessary for bacterial growth [3]. Once inside the textile, microorganisms can form biofilms and cause serious concerns, including fabric rotting, staining, unpleasant odors, and health concerns ranging from simple discomfort to physical irritation, allergic sensitization, toxic responses, infection, and disease [3,4].

Microorganisms residing in biofilms are embedded in a self-produced layer of extracellular polymeric substance (EPS) [5,6] that protects bacteria against detrimental environmental conditions such as UV irradiation, antibiotics, and disinfection, which makes them much more tolerant compared to planktonic bacterial cells [7,8,9]. Likewise, EPS impedes accessibility of the bacteria to antimicrobials, which increases resistance to the cleaning and sanitizing chemicals usually employed for their removal and/or prevention [7,10,11].

Microbial growth and biofilm formation in clothes and fabrics are very common problems because of sweating and wet conditions. Interaction of textiles with pathogenic and nonpathogenic microorganisms can lead to the outcomes discussed in [4]. The interaction of microorganisms with textiles is a complex process which depends on various factors such as the type of microorganism, surface characteristics of microbes, various environmental factors (physical and chemical), and physicochemical characteristics of the textile surface [12,13,14]. The microorganisms reversibly adherent to textiles can be liberated and redispersed into the air or liquid (e.g., during washing) and thereby transferred to the surrounding environment through direct or indirect contact, providing a significant cross-contamination or cross-infection potential [15,16], which is especially critical in hospital settings, where nosocomial infections are a serious threat to human health.

There is clinical evidence of a close relationship between environmental hygiene and transmission of microorganisms which produce nosocomial infections, such as environmental contamination [17]. According to The Worldwide Outbreak Database [18], the following bacteria play major roles in outbreak events: *Staphylococcus aureus*, *Klebsiella pneumoniae*, *Pseudomonas aeruginosa*, *Acinetobacter baumannii*, *Serratia marcescens*, *Enterococcus faecium*, *Escherichia coli*, and *Enterobacter cloacae*. Among those, *P. aeruginosa* is one of the major opportunistic human pathogens that can cause pneumonia, bloodstream infections, urinary tract infections, and surgical site infections, and is particularly dangerous for immunocompromised patients. *P. aeruginosa* has earned the name of an opportunistic pathogen that forms a biofilm on different surfaces and is responsible for 10–20% of infections in hospitals [19]. Their pathogenicity depends on various virulence factors, including cell-associated factors, i.e., flagella and pili, acting as adhesins and motility factors, as well as adherence and biofilm formation abilities on both biotic and abiotic surfaces and the production of lipopolysaccharides [20]. This makes *P. aeruginosa* a significant model organism for investigating the development of bacterial biofilms and resistance to various antibacterial agents [21]. Further, many extracellular virulence factors play an important role in the pathogenicity of *P. aeruginosa*, such as the secretion of proteases, lipases, phospholipase, elastases, and exotoxins; the production of pigments such as pyocyanin; and the production of quorum-sensing molecules [22].

The ability of biofilm-resident bacteria to survive with minimal nutrient requirements and greater tolerance to numerous physical conditions enables them to persist in both urban and natural settings. The biofilm lifestyle further provides basic mechanisms of resistance not only to antibiotics but also to bacteriophages, disinfectants, and other host defense systems [23] by constituting multilayered protection mechanisms [24].

Various methods are used to wash fabrics and remove biofilms. The antimicrobial effect on textiles is achieved via “biostatic” approaches, which inhibit cell growth, or “biocidal” approaches that promote killing of pathogens [25]. Antimicrobial agents that have been used in textiles up to now include quaternary ammonium compounds (QACs), N-halamines, chitosan, polybiguanides, triclosan, nanoparticles of noble metals and metal oxides, and bioactive plant-based products. QACs are also used as detergents, softening agents, or antistatic agents at different stages of textile processing such as pretreatment, dyeing, and finishing [26,27]. Although these compounds are very efficient at eradicating bacteria in suspension, bacteria residing inside a biofilm are often less sensitive due to their protective EPS, which impedes accessibility of the bacteria to the antimicrobial. Especially for textiles, it is essential not only to focus on bacterial killing, but also on removing the remaining EPS that otherwise facilitates colonization by other bacteria.

In textile applications, such as clothing, coatings with antimicrobial agents are used as one preventive strategy that should provide effective protection from a wide variety of pathogens. Agents must be durable to washing, dry cleaning, and ironing, be simple and easy to apply on textiles, and should not compromise the appearance and hand quality of textiles [28,29]. Bridier et al. [30] suggest that an efficient formulation may therefore be composed of mixtures of enzymes for different substrates to destabilize the EPS, such as proteases, cellulases, polysaccharide depolymerases, alginate lyases, dispersin B, and DNases. Enzymes have been proven effective for the degradation of the EPS of biofilms [31,32]. The enzymes destroy the EPSs by degrading their physical integrity, and the efficiency will depend on their composition [33].

Considering the problem of biofilms on textiles and the current limited removal strategies, this study reports on the specific combination of a formulation based on detergent and enzymatic activity for the removal of *P. fluorescens* and *P. aeruginosa* biofilms that served as test model organisms on cellulose. To screen for efficient formulations for biofilm removal, a reliable standardized biofilm model is needed, which is suitable to investigate the removal of biofilms in a reproducible manner using soft fabrics as a model substrate. The biofilm model platform should offer optimal growth conditions and be easy to handle in a daily lab routine for standard screenings, as commonly used biofilm reactors and biofilm models [34] are not easily transferable to a cellulose fabric biofilm model. Therefore, this study used a tailored biofilm growth chamber platform.

Standardization is still a challenging matter as biofilms are living, complex, highly heterogeneous, and constantly evolving structures. Most researchers have employed the conventional direct plate count method (colony forming unit (CFU) determination) for the quantification of adherent bacterial cells on different biomaterials, or used metabolic colorimetric dyes, ATP bioluminescence, or propidium monoazide-qPCR [35]. The most widely used technique to quantify the viable biomass of a surface is to determine the CFU on agar media, after biomass detachment from the surface [34].

Catto and Capitelli [36] summarized approaches to study the EPS matrix, such as fluorescence microscopy, spectroscopic techniques, protein stains, or polysaccharide quantification. Recent studies [37,38,39,40] reported on novel methods to evaluate biofilms and their metabolites without destroying the EPS matrix, such as scanning Kelvin probe (SKP), Fourier transform infrared microscopy (FTIR), digital holographic tomography (DHT), Raman spectroscopy, surface-enhanced Raman scattering (SERS) spectroscopy, and liquid microjunction surface sampling probe accoupled to mass spectrometry. To identify the spatial distribution of bacteria residing in biofilm, FISH (fluorescence in situ hybridization)-based techniques and immunofluorescence are used [34]. To analyze the biofilm morphology, including its three-dimensional spatial distribution, confocal laser scanning microscopy (CLSM), fluorescence recovery after photobleaching (FRAP), fluorescence correlation spectroscopy (FCS), and fluorescence lifetime imaging microscopy (FLIM) are used [41,42,43,44]. Conventional electron microscopy is widely used to achieve imaging at subnanometer resolution, providing a detailed insight into the ultrastructure of the biofilm and its environment [45]. It is also possible to employ scanning electron microscopy (SEM), transmission electron microscopy (TEM), cryo-SEM, environmental scanning electron microscopy (ESEM), focused ion beam (FIB)-SEM, atmospheric SEM (ASEM), and super-resolution microscopy (SMR) [4,34,35]. To evaluate the mechanical and physical properties of biofilms, atomic force microscopy (AFM), AFM single-cell force spectroscopy (SCFS), quartz crystal microbalance (QCM), and rheometry are used [46,47,48].

For solid and stable substrates (i.e., not fabrics), various methods have been reported to study biofilms or different components of biofilms. Quantification methods include those assessing only viable biomass, those able to detect both live and dead cells, as well as techniques investigating the whole biofilm, including both cellular and EPS components. Indeed, which method is the most appropriate to be chosen highly depends on the type of material [36].

Cellulose-based fabrics come with various challenges to monitor biofilm removal, as for soft material, complete removal of bacteria via physical methods and CFU counting makes it necessary to adapt standard procedures.

Light and fluorescence microscopical methods are further limited for fabrics because they are mostly not transparent or have an uneven structure, and dyes to stain bacteria for microscopy use to adsorb to cellulose (own tests). Furthermore, other methods to remove and disintegrate biofilms to singularize bacteria for CFU counting have to be adopted for fabrics, but also alternative biochemical methods are not appropriate, such as the standard crystal violet assay [49] to stain the adherent biomass on certain biofilm substrates, such as cellulose, because they are strongly stained by crystal violet [50].

In the context of their research on textiles, colony biofilm assays allowed Tran et al. [51] to successfully examine the effectiveness of cellulose discs coated with organoselenium in inhibiting *P. aeruginosa* and *S. aureus* in biofilm-related wound infections. Bajpai et al. (2011) [35] uncovered the mechanism of adherence of *E. coli* on different textiles using SEM and FTIR spectroscopy. Rajkowska et al. (2019) [52] studied the virulence attributes of *P. aeruginosa* isolated from pre-Columbian textiles and compared them to clinical strains employing CFU counting methods, SEM microscopy, SEM-EDX, and FTIR analyses.

Based on the literature and taking into account the special characteristics of the evaluated cellulose substrate, this study aimed to monitor biofilm growth of both *P. fluorescens* and the opportunistic pathogen *P. aeruginosa* on cellulose fabrics and their removal using either detergent- or enzyme-based formulations separately or in combination. Likewise, we reported for the first time, according to our knowledge, on a novel tailored biofilm model for fabrics. We integrated both diverse microscopic techniques (SEM, FE-SEM, 3D laser microscopy, epifluorescence, QCM) to visualize biofilm removal efficiency and quantification of biofilm-residing living bacteria, which could successfully overcome the abovementioned technical challenges of biofilm monitoring on cellulose fabrics.

## 2. Materials and Methods

### 2.1. Often Used Materials

Milli-Q water was drawn from a Millipore Direct Q8 system, with a resistivity of 18.2 MΩ cm (Millipore advantage A10 system, Schwalbach, Germany, with Millimark Express 40 filter, Merck, Darmstadt, Germany). A phosphate saline buffer solution (PBS, pH 7.4) was prepared through the dilution of DPBS by Milli-Q water, and the volume ratio of DPBS and Milli-Q water was 1:9.

### 2.2. Bacterial Growth

Unless mentioned otherwise, the Luria–Bertani (LB) medium was used. The bacterial glycerol stock was stored at −80 °C. *P*. *aeruginosa* GFP (ATCC 10145GFP) and *P. fluorescens* (ATCC 13525) were grown overnight at 28 °C and 37 °C, respectively, for 24 h, from a single colony in 5 mL of LB broth—in the case of *P. aeruginosa* GFP, this was supplemented with ampicillin (100 μg/mL)—in a shaking incubator (MaxQ 4000 benchtop orbital shaker, Thermo Scientific, Waltham, MA, USA) at 120 rpm. Then, the bacteria suspension was diluted to 1:100 (OD600 ≈ 0.5).

### 2.3. Substrate

Cellulose-based woven substrates of 15 or 32 mm in diameter were used to grow *Pseudomonas* spp. biofilms. Autoclaved cellulose (15 min at 121 °C and 1.2 bar) was used for the biofilm models.

### 2.4. Biofilm Growth

**Figure 1:**
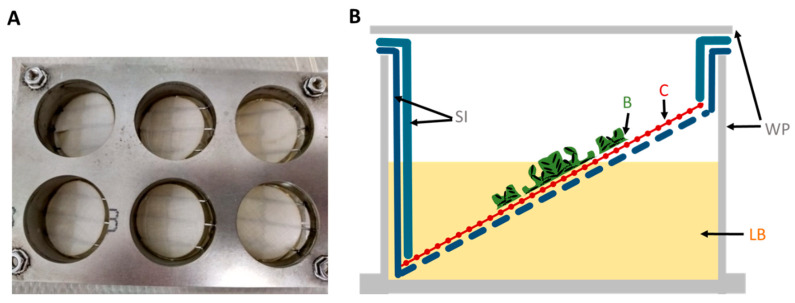
Setup of the stainless steel 6-well plate growth platform to grow *Pseudomonas* biofilms on fabrics. (**A**) Photograph from top of the 6-well stainless steel insert that clamps the cellulose woven substrate. (**B**) Schematic of one 6-well plate from the side view. LB covers only half of the substrate, so that the *Pseudomonas* biofilm can grow at the liquid–air interface. WP—well plate; LB—culture media; B—*Pseudomonas* biofilm; C—cellulose substrate; SI—stainless steel inserts.

The autoclaved cellulose was placed into a stainless steel 6-well plate (SS WP) growth chamber platform specifically designed by BASF SE and reproduced by the workshop of the University of Siegen for this experiment (Figure 1). LB covered only half of the surface of the tilted substrate to create a liquid–air interface where biofilms grow best. The nutrient supply offered from both above and below in the SS WP optimized the growth of *Pseudomonas* spp. biofilms on woven fabrics, which guarantees optimal nutrient supply while fixing the fabric at the liquid–air interface.

In total, 5 mL of *P. aeruginosa* or *P. fluorescens* suspensions in LB and their respective control (LB media without bacteria) were placed in each SS well. The biofilm model was sterile covered and incubated at 120 rpm for 48 h at 28 °C or 37 °C for *P. fluorescence* and *P. aeruginosa*, respectively. LB media was renewed with fresh LB media every 24 h.

For biofilm growth for QCM measurement, standard gold-coated quartz sensors were cultured with 300 µL of *P. fluorescence* suspension in 24-well plates at 28 °C for 2 days at 120 rpm, tilted at 45° in the incubator holders so that a liquid–air interface allowed optimal biofilm growth on the substrate, and with a media exchange after 24 h. After removing the growth media and washing the QCM samples with PBS (gently shaking for 1 min to remove unattached bacteria), they were fixed at room temperature in 2.5% glutaraldehyde in PBS for 24 h, washed again 3 times with PBS, and stored in PBS at 4 °C until QCM measurement was performed.

### 2.5. Treatment for Biofilm Removal

Three solutions were used to remove the biofilm formation on cellulose-based woven textiles: (1) Formulation 1 (F1), detergent-based; (2) Formulation 2 (F2), enzyme-based; and (3) Formulation 1/2 (F1/2), a combination of both detergent and enzymes. The active substances of all formulations used were dissolved in hard water (14 °dH). Cellulose in the wells incubated for 48 h in a biofilm model (with *Pseudomonas* species or LB media as control) was removed and rinsed three times with PBS to eliminate the rest of the LB media or remove unattached bacteria. Thereafter, the sample was immersed in 2 mL of biofilm removal formulations (F1, F2, or F1/2) with PBS as control for 1 h at room temperature and 200 rpm. Before biofilm readout, the wells were washed three times with PBS to eliminate the rest of the biofilm removal formulations. Finally, all liquid was removed before immediately applying the specific quantification method.

### 2.6. Quantification of Remaining Living Bacteria on the Textile Fabrics (CFU Counting)

Based on the previous results regarding the best-performing formulation, viable bacteria of *P. fluorescens* were quantified comparing the control condition with the biofilm on the cellulose treated with F1/2. In this assay, 15 mm diameter celluloses were used, and they were inoculated in a tilted plastic 24-well plate.

To determine the composition of the remaining surface-attached bacteria, the cell number was estimated by placing the cellulose (diameter: 15 mm) into a glass test tube with PBS and five glass beads. Each suspension was sonicated once for 2 min at 25 °C, at 100% intensity, (Bandelin sonorox digital plus) and vortexed at full speed for 2 min, following previously reported protocols [53,54]. Then, samples were serially diluted with PBS and counts determined according to the plate count agar method. Samples were incubated for 24 h at 37 °C. Cellulose substrates were tested in triplicate under identical conditions. The results were expressed as CFU/cm^2^.

### 2.7. SEM and FE-SEM

After the cellulose was washed with PBS, biofilms on cellulose were fixed at room temperature in 2.5% glutaraldehyde in PBS and stored for 24 h at 4 °C. The washing with PBS step was repeated, and the cellulose coupons were dehydrated in serial dilutions of 30, 50, 60, 70, 80, and 90% ethanol for 10 min each, followed by three rinses for 10 min in 100% ethanol. For the final complete drying, 100% hexamethyldisilazane (HMDS) was used. Cellulose coupons were immersed in HMDS twice for 30 s and air-dried for 10 s. The samples were sputter-coated with a thin gold layer (8–10 nm) in a sputter coater (S150B, BOC Edwards, UK) at a pressure of 0.2 mbar in argon atmosphere for 2 min, at a voltage of 1.0 kV. SEM analysis was performed with the CamScan 24 SEM, 1990 (Electron Optics Instruments, West Orange, NJ, USA). The FE-SEM (Zeiss Ultra 55cv, ZEISS, Oberkochen, Germany) was operated with a maximum voltage of 26 kV. All measurements were performed with an operating voltage of 5.0 kV, and the detector was the Inlens secondary electron detector. At minimum, biofilms grown on two cellulose samples in two independent experiments were analyzed using microscopy techniques.

### 2.8. Three-Dimensional Laser Scanning Microscopy

The 3D laser scanning microscope LEXT OLS4000 (Olympus, Hamburg, Germany) was used to analyze the effect of the F1/2 formulations on biofilms of *P. aeruginosa* based on the samples prepared in Section 2.7.

### 2.9. Epifluorescence Microscopy

Cellulose substrates with a *P. fluorescens* biofilm that exhibited an autofluorescence were subject to fluorescence analysis using a Zeiss Axiovert 200 with 4-fold magnification and the following filter set: Ex., 450–490 nm; and Em., 500–550 nm.

### 2.10. QCM Measurements

For the QCM experiments, fixed biofilms were immobilized on standard gold-coated quartz sensors (QSX 301 Au, Biolin Scientific, Gothenburg, Sweden), as described above in the “Biofilm Growth” section. The resulting biofilm-covered sensors were mounted in the flow cells of a Q-Sense E4 instrument from Biolin Scientific, which was equipped with a custom-designed flow-through system operated at 23 °C and a flow rate of 50 µL/min. Biofilms fixed on 2 independent quartz sensors were analyzed via QCM. As a reference, biofilm-free gold-coated sensors were used as substrates in the same way. All sensors were first exposed to a flow of pure deionized water (taken from a Milli-Q Advantage A10 water purification unit) until stable baseline readings in resonance frequency and dissipation were achieved. After another 30 min, the system was switched to hard water (14 °dH, pH 8.5), which in all cases led to a slight mass increase due to ion adsorption before stable baseline readings were again established. Subsequently, the substrates were incubated for 60 min with detergent-based F1 in hard water, followed by another 60 min treatment with a solution containing both the enzyme and detergent (F1/2) in hard water. After exposure to F1/2, the substrates were rinsed successively with detergent-containing F1 in hard water for 30 min, hard water without detergent for 30 min, and finally pure deionized water for 10 min. During all stages, changes in resonance frequency (ΔF) and dissipation (ΔD) were measured continuously at intervals of ≈1.5 s for all available overtones (*n* = 3, 5, 7, 9, 11, 13) of the main resonance. After the QCM experiment, the biofilm-coated sensors were dried in air (after QCM analysis) and characterized by optical and scanning electron microscopy (SEM) using a Nikon Eclipse LV100ND and a Phenom proX microscope, respectively. Before SEM, imaging was performed at 10 kV after previously coating the substrates with a thin layer of sputtered gold. The control fixed biofilm on QCM samples, which were not gold sputtered, were analyzed via SEM as described in the method section “SEM and FE-SEM” using the CamScanSEM (CS24, Applied Beams, Beaverton, OR, USA).

### 2.11. Statistical Analysis

Cell counts were converted to decimal logarithmic values (log CFU/cm^2^) to nearly match the assumption of a normal distribution. In all analyses, triplicate tests were performed under identical conditions in two independent experiments, and the results were expressed as means and standard deviations (mean ± SD). Student’s *t*-test was used for comparison of 2 treatment groups. A confidence level equal to or higher than 95% was considered statistically significant (* *p* < 0. 05, ** *p* < 0.01, *** *p* < 0.001).

## 3. Results and Discussion

**Figure 2:**
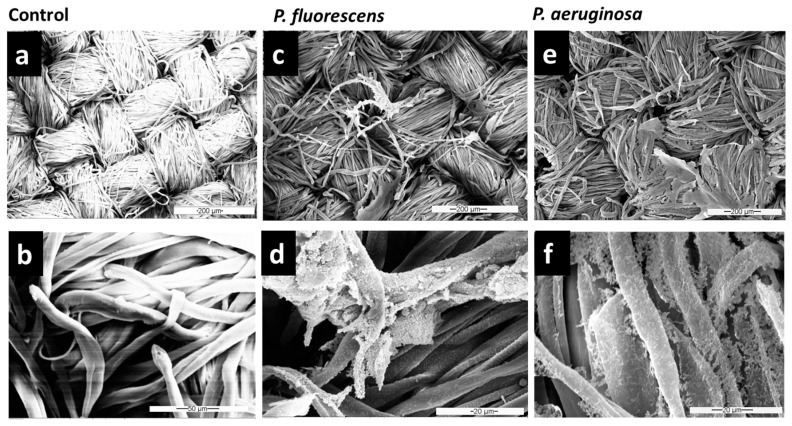
SEM images of autoclaved cellulose incubated with LB only (without bacteria) as control (**a**,**d**) and biofilm formation of *P. fluorescens* (**c**,**d**) and *P. aeruginosa* (**e**,**f**) grown for 48 h. Lower (**a**,**c**,**d**) and higher (**b**,**d**,**e**) magnifications are shown.

Cellulose is known worldwide as the most abundant, renewable, and an almost inexhaustible polymeric raw material with fascinating chemical structure and properties [55]. To use this substrate for the biofilm model on textiles, we needed to guarantee its sterility. It was observed via SEM analysis that cellulose fibers stayed intact upon autoclaving (Figure 2a,b), and their morphology and microstructure did not change due to the autoclaving process in comparison to untreated cellulose.

The autoclave cellulose was selected and used for studying the biofilm growth of *P. aeruginosa* and *P. fluorescens* in the fabric biofilm model. Figure 2d,f show that *P. fluorescens* and *P. aeruginosa* could adhere strongly to the textile surface and form dense networks on this inanimate surface. Bacteria were distributed all over the fibers, establishing central microcolonies (clusters of cells) embedded in the self-produced EPS.

Varshney et al. [13] studied different bacteria strains on textile materials, and among the four bacteria studied, the bacterial count of *P. aeruginosa* was highest (2.79 × 10^8^ CFU/mg), underlining that this species is a strong biofilm producer. They reported that the bacterial load on fibers is highly dependent on both the type of bacteria and the fiber. *P. aeruginosa* exhibited low adhesion to cotton, polypropylene, silk, and wool, with intermediate adhesion to viscose, and the highest adhesion to polyester [13]. The binding ratios of *S. aureus* and *P. aeruginosa* were compared on cotton, polyester, acrylic, nylon, and wool fibers [56]. It was found that *P. aeruginosa* bound to polyester and acrylic fibers at the highest ratio (99.9% and 95.4%), to wool at an intermediate ratio (84.7%), while the least binding ratio was observed on nylon and cotton fibers (14.9% and 8.1%). Interaction of fiber surface with single as well as multiple layers of bacteria was also observed. The type of fiber may also influence the clumping of cells. In our study, it was observed that bacteria did not cover the surface of fibers uniformly. Related results of bacterial adhesion were obtained at the fabric level through SEM analysis, which gave only a qualitative assessment of adhesion on different fibers [13,35,57].

**Figure 3:**
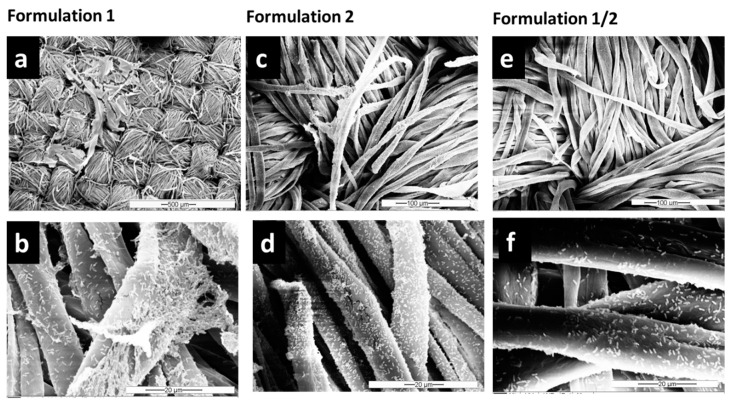
SEM images of *P. fluorescens* grown for 48 h to form biofilms on cellulose substrates after 1 h of washing with Formulation 1 (detergent-based (**a**,**b**)), Formulation 2 (enzyme-based (**c**,**d**)) and the combination of both (Formulation 1/2 (**e**,**f**)). Lower (**a**,**c**,**e**) and higher (**b**,**d**,**f**) magnifications are shown.

Since microorganisms residing on textiles can have several harmful effects, it is important to avoid microbial growth or to remove established biofilms from the material. Many conventional mechanical and chemical routine methods are available to contain biofilm formation in different industrial equipment [58], but these methods do not work for every setting where reliable removal is needed, such as for biofilm removal from various types of fabrics. Therefore, in this study, once it was observed that *Pseudomonas* species adhere strongly to the cellulose surface, different washing formulations (based on detergent and enzymatic solutions) were tested to remove biofilms of *Pseudomonas* spp. from textiles. SEM images (Figure 3 and Figure S1) showed that the combination of both detergent and enzymes in F1/2 exhibits the highest efficiency in *P. fluorescens* biofilm removal. After treatment with F1/2, only single bacteria or residuals of EPS were still attached along the cellulose fibers (Figure 3e,f). The treatment with the detergent-based formulation F1 was the least effective of the three formulations (compare Figure 2c,d and Figure 3e,f).

Furthermore, a third experimental reproduction of the effective biofilm removal of F1/2 was proven by a biofilm model using cellulose substrates with smaller diameter inoculated in a tilted plastic 24-well plate. Via FE-SEM (Figure S2), it was possible to demonstrate the ultrastructure of *P. fluorescens* microcolonies on the fibers. The control sample clearly revealed a cluster of cells connected by fibrils, which are supposed to be pili (Figure S2, red arrow). In contrast, biofilms treated with F1/2 exhibited a reduction of *P. fluorescens* adherent to the fibers, which were isolated and not in the content of microcolonies.

**Figure 4:**
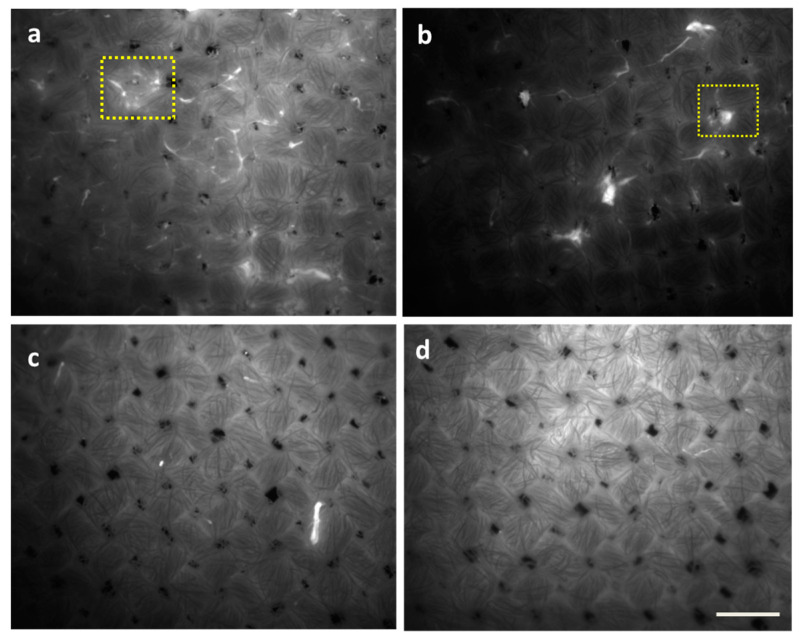
Epifluorescence microscopy images of *P. fluorescens* grown for 48 h to form biofilms on cellulose substrates after 1 h of washing with detergent-based Formulation 1 (**b**), enzyme-based Formulation 2 (**c**), and the combination of both in F1/2 (**d**). Untreated biofilms are shown in (**a**). Bright spots (indicated by yellow boxes as examples) indicate biomass due to bacteria aggregates and biofilm attachment. Scale bar: 200 µm.

Additionally, the effect of the tested formulations on the *P. fluorescens* biofilm was evidenced by epifluorescence microscopy and CFU agar plate counting. Through epifluorescence microscopy (Figure 4), based on the autofluorescence of *P. fluorescens*, the effect of F1/2 on biofilm removal was confirmed. Furthermore, it was revealed that detergent-based F1 exhibits the lowest removal efficiency (Figure 4b), followed by enzyme-based F2 (Figure 4c), while the combination F1/2 (Figure 4c,d) exhibited a better biofilm removal efficiency.

**Figure 5:**
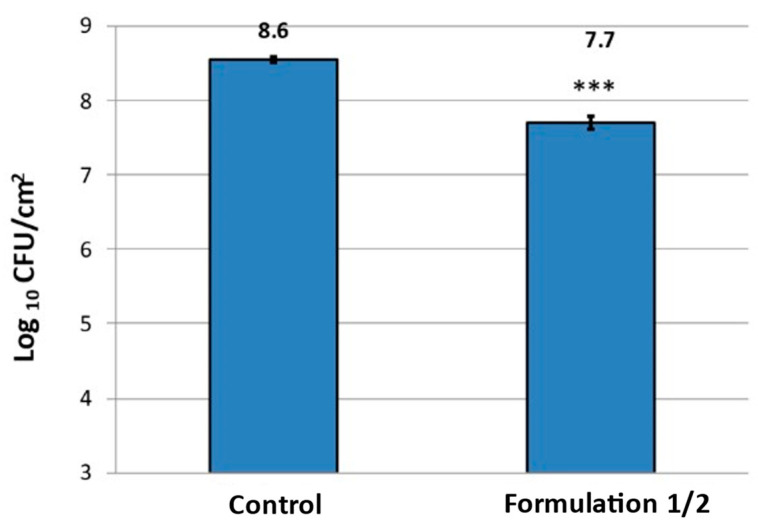
Reduction of *P. fluorescens* on the cellulose substrate after treatment for biofilm removal. *P. fluorescens* grown for 48 h to form biofilms on cellulose substrates were washed for 1 h with Formulation 1/2 or not treated (control). Bacteria were extracted from biofilms on the cellulose substrate via mild ultrasonication. Resulting bacteria suspensions were serially diluted, and the concentration of viable bacteria was determined via colony forming unit counting. Concentration of viable *P. fluorescens* is expressed as mean log CFU/cm^2^ ± SD. *** *p* < 0.001 (*n* = 3).

Supporting this data, it was proven that a significant reduction (*p* < 0.001) of viable cells could be achieved after washing with formulation F1/2, which was reduced around one log in comparison with the control (Figure 5).

In summary, when considering the absolute reduction of viable bacteria on cellulose and the microscopically visible reduction of *P. fluorescens* biofilms, F1/2 proved to have a significant biofilm removal effect. However, several viable single bacteria remained on the substrate.

**Figure 6:**
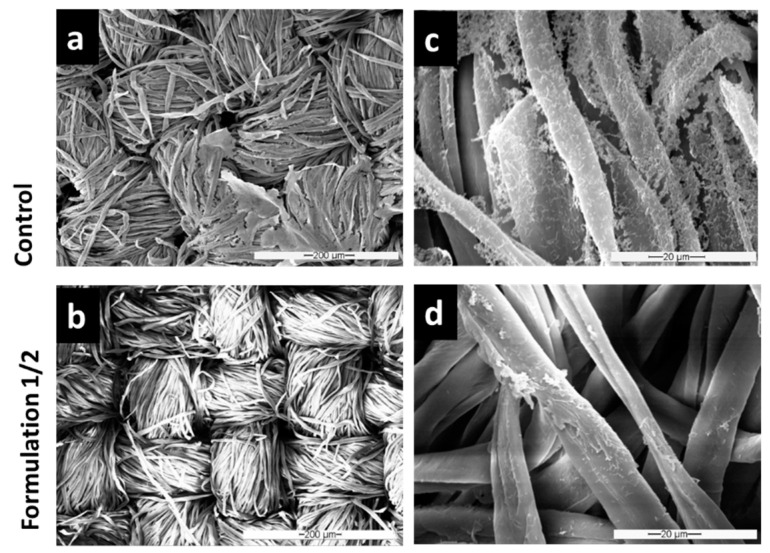
SEM images of *P. aeruginosa* grown for 48 h to form biofilms on cellulose substrates after 1 h of washing with Formulation 1/2 (**b**,**d**) in comparison to untreated controls (**a**,**c**).

**Figure 7:**
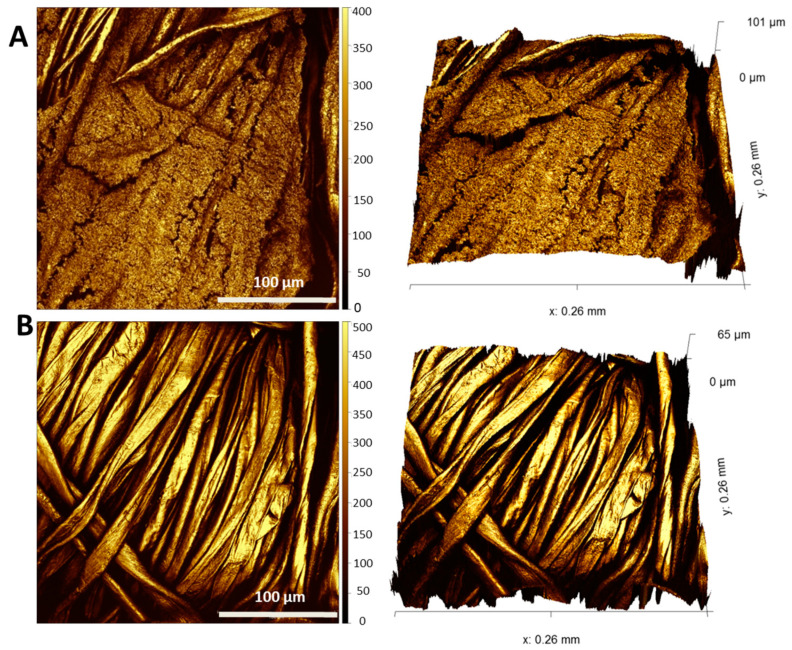
The 3D laser scanning microscopy images of *P. aeruginosa* grown for 48 h to form biofilms on cellulose substrates after 1 h of washing with F1/2 (**B**) in comparison to untreated controls (**A**). The pictures on the right side are tilted 3D illustrations.

To prove that this removal routine is also effective against the more health relevant opportunistic pathogen *P. aeruginosa*, we investigated biofilms grown in our textile biofilm model, with and without biofilm removal treatments, microscopically via SEM and 3D laser scanning microscopy. It is apparent from Figure 6 and Figure 7 that the biofilm of this pathogenic bacterium was reduced with even higher efficiency by F1/2 than *P. fluorescens* was.

Although *P. aeruginosa* biofilms seemed to exhibit an even higher fiber coverage and layer thickness in the control condition (Figure 6a,c and Figure 7A) than *P. fluorescens* biofilms, there were even less residual single bacteria or biomass found after exposure with F1/2, as illustrated by the clean fibers in both Figure 6b,d and Figure 7B.

To gain further insights into the interactions between different washing formulations and biofilm surfaces, in situ QCM measurements were performed. With quartz crystal microbalance (QCM), interactions between liquid formulations and a surface (e.g., adsorption, swelling, dissolution) can be monitored online by tracing time-dependent changes in resonance frequency and dampening behavior (dissipation) of a quartz crystal resonator, which is covered by the substrate of interest and exposed to the respective formulation in flow-through mode [59]. Results obtained for a typical reference experiment on a blank gold substrate without attached biofilm are shown in Figure S3. The observed changes in resonance frequency (Figure S3a) indicate fast adsorption of the detergent in F1 on the Au surface (step between Stages 1 and 2). Upon addition of enzyme-containing F2 (Stage 3), no additional modifications occur, suggesting that already adsorbed surfactants inhibit binding of the enzyme to the surface. Upon final rinse with hard and deionized water (Stages 5 and 6), partial desorption of the surfactants takes place, indicating that their adsorption on Au is reversible under the chosen conditions.

Given that the time-dependent profiles of the different frequency overtones in Figure S3a overlap and minor (few ppm) concurrent changes in dissipation are observed in Figure S3b, the formed surfactant adlayer can be considered as rigid with negligible viscoelastic contributions, allowing the Sauerbrey equation to be used for conversion of frequency into mass changes [60]. Corresponding calculations yield an adsorbed surfactant mass of ca. 160 ng/cm^2^ in Stage 2. Assuming an average area per surfactant headgroup of 30 Å^2^ and an average molecular weight of 300 g/mol, the determined adsorbed mass agrees well with the formation of a surfactant monolayer (expected Δm = 166 ng/cm^2^).

**Figure 8:**
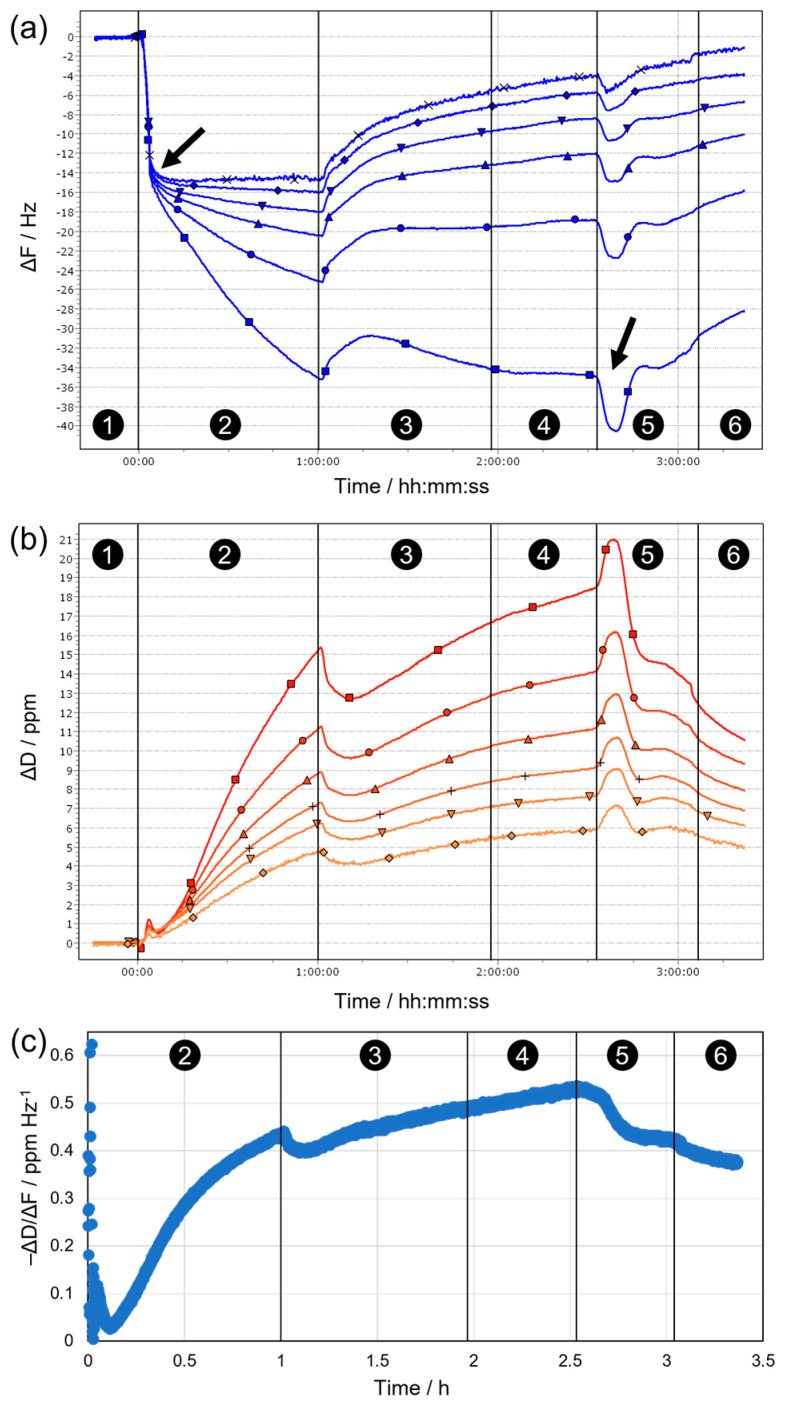
Effects of surfactants and enzymes on fixed *P. fluorescens* biofilms, as monitored in situ by QCM. (**a**,**b**) Time-dependent changes of (**a**) frequency and (**b**) dissipation during sequential incubation of a fixed biofilm (immobilized on a gold-coated QCM sensor) with (1) hard water, (2) detergent in hard water (F1), (3) enzymes and detergent in hard water (F1/2), (4) detergent in hard water (F1), (5) hard water, and (6) deionized water, shown in an exemplary result of 2 independent QCM measurements of separate QCM sensors with fixed biofilms. Arrows indicate initial dip in ΔF after ca. 3 min and second prominent dip after 2.5 h. For both ΔF and ΔD, signals from different overtones (n) of the main resonance are shown (squares: *n* = 3, circles: *n* = 5, triangles: *n* = 7, inverted triangles: *n* = 9, diamonds: *n* = 11, crosses: *n* = 13). (**c**) Plot of the ratio of dissipation and frequency changes over time for the third overtone, representing a measure for the mass-normalized softening of the biofilm surface.

The behavior observed during the same sequence of stages on sensors covered with fixed biofilms is fundamentally different (Figure 8). This becomes evident already upon initial contact of the substrate with water, where a slight mass loss occurs (due to release of loosely adhering parts of the biofilm), which is followed by a slow mass gain (due to swelling of the remaining biofilm), until eventually a stable baseline is established in hard water (Stage 1 in Figure 8b). After switching to F1 (Stage 2), a rapid initial decrease in resonance frequency takes place, indicating the formation of a surfactant (mono)layer on the biofilm surface, which appears to be rigid as on the blank Au substrate, as suggested by overlapping frequency overtones (Figure 8a) and minor dissipation changes (Figure 8b) during this period (completed initial adsorption is indicated by the arrow after ca. 3 min in Figure 8a). Subsequently, the frequency decreases further at a slower rate, and the different detected overtones begin to spread towards the end of Stage 2. In parallel, the dissipation increases significantly, with significant differences between overtones. This behavior proposes non-negligible contributions from viscoelastic effects, i.e., the biofilm surface and/or the formed surfactant adlayer become soft(er), for example, owing to stronger hydration/swelling or progressive disintegration of the biofilm (therefore, the higher overtones show less pronounced changes due to their lower penetration depth into the sample). Upon introduction of F1/2 (Stage 3), all frequency overtones start to increase while a rather sharp drop in dissipation is observed—both indicate partial removal of the biofilm via enzymatic action. This trend continues (except for ΔF3) throughout Stage 3 and extends until the end of Stage 4, as rinsing with enzyme-free, detergent-containing F1 means that the enzymes remain at the surface and continue the breakdown of neuralgic points in the biofilm even when the bulk solution is replaced. Removal of surfactants from the bulk, i.e., rinsing with hard water in Stage 5, first induces a dip in ΔF (indicated by the arrow at ca. 2.5 h in Figure 8a) and a concurrent peak in ΔD, before the previous trend of slow biofilm removal resumes and continues to the end of Stage 6 (rinsing with deionized water). The minimum in ΔF and maximum in ΔD suggest a temporary mass increase at the surface, possibly caused by the collapse of a loosely adhering, strongly hydrated outer surfactant layer when the interfacial tension with the bulk medium is suddenly increased. After such a kinetically controlled collapse, the excess surfactant desorbs from the surface until a (meta)stable last layer remains, and proteolytic degradation can proceed.

In general, the dissipation signal can be regarded as a measure of the dampening of the sensor oscillations and thus the “softness” of the surface. However, any increase in mass at the sensor surface (e.g., by adsorption of a rigid surfactant layer) will also cause enhanced dampening and higher dissipation, even though no net softening occurs. Therefore, we chose to plot the ratio of dissipation and frequency changes (−ΔD/ΔF) as a measure for the “mass-normalized” degree of softening during sequential biofilm treatment, as shown for n = 3 in Figure 8c. It is evident that initial incubation with F1 (Stage 2) leads to substantial net softening, likely because of surfactant penetration into the biofilm, accompanied by uptake of water and strong swelling. This trend is continued at a slower rate in Stages 3 and 4, where concurrent partial removal of the biofilm by the addition of enzymes in F1/2 competes with surfactant-induced swelling. Finally, upon replacement of detergent-containing F1 by hard and deionized water (Stages 5 and 6), the apparent degree of softening decreases again as surfactants desorb from the biofilm and swelling effects are reversed.

Due to the spreading of overtones observed on the biofilm surface for both frequency and dissipation changes, the classical Sauerbrey equation can no longer be applied to calculate layer thicknesses. Instead, viscoelastic modeling of the data was performed using the QTM software [61], which analyzes the overtone dependence of ΔF and ΔD. Results obtained in this way for the state of the surface at the end of each of the stages are summarized in Table 1.

The obtained net adlayer thicknesses (d, all referenced to the state in hard water during Stage 1) confirm the qualitative considerations made above, in a sense that surfactant ad/absorption and biofilm removal through enzymatic action compete. After final rinsing with hard water (e.g., regarding the difference between Stage 5 and Stage 1, in which the bulk solution conditions are identical), a net gain of ca. 15 nm is observed due to residual excess surfactant. The simultaneously determined viscoelastic properties (e.g., the storage (G′) and loss (G″) moduli) of the formed adlayer reveal viscous-dominated behavior (G″ > G′) in all stages, indicating considerable hydration, which also becomes manifest in the derived viscosity values that are only 20–30% higher than pure water at the end of Stage 2. During the subsequent stages of treatment, the elastic contribution gradually increases, which may be caused by increasing amounts of enzymes in the biofilm and/or by the ongoing removal of softer parts of the biofilm caused by enzymatic cleavage. In turn, the viscosity remains low and “water-like”, suggesting persistently high hydration. Taken together, these observations indicate a complex, and partly opposing, interplay between surfactants and enzymes in the removal or modification of fixed biofilms from surfaces.

Under the chosen conditions, biofilm removal is anything but complete, as proposed by the in situ QCM data and confirmed by ex situ characterization using electron and light microscopy (Figure S4). The net coverage of the sensor with biofilm is indeed similar after the QCM experiment (Figure 3b,c) compared to the state before (Figure S4a). However, individual structures are more defined before treatment, whereas afterward, various filamentous networks and structures with cloudy appearance are observed (cf. Figure S4b), possibly as a combined result of surfactant adsorption, partial enzymatic breakdown, and aggregation/coalescence. Incompleteness of removal may also be caused due to the fixation of the biofilm that has to be performed for QCM measurement for biological safety reasons. The fixation does not only inactivate the bacteria, but it also causes a stronger attachment of the biofilms, which might show less efficient removal than with unfixed living biofilms.

Enzymes can destabilize the EPS by destroying the physical integrity of the EPS [33]. This approach can therefore circumvent the disadvantage of other antimicrobial agents that fail to penetrate the biofilm due to the EPS, which acts as a protective barrier.

Not only the is inactivation of bacteria, proved in this study with CFU counting, essential to contain biofilm growth on textiles, but the attached biofilm mass must also be removed because the inactivated bacteria may provide an ideal environment for further adhesion and growth, resulting in other potentially pathogenic microorganisms.

Molobela et al. [62] evaluated the eradication of *P*. *fluorescens* biofilms using protease (savinase, everlase, and polarzyme) and amylase (Amyloglucosidase and bacterial Amylase Novo). All enzymes tested except for the protease Polarzyme were effective for the degradation of the biofilm EPS. The way the enzymes degrade the proteins in the EPS is through binding and hydrolysis of the protein molecules and converting them into smaller units that can be transported through the cell membranes and then be metabolized.

To summarize, our established textile biofilm model proved gradual efficiencies of biofilm removal using the three different formulations tested. It was possible to demonstrate in a reproducible way with both microscopical and quantitative readouts that the alternative removal approach proposed here, a combination of detergent with enzymatic activity, offers a successful strategy to treat and clean textiles that are contaminated with robust *Pseudomonas* spp. biofilms. Combining enzyme- and detergent-based agents could be a useful strategy for routine treatment of textiles that is able to both destabilize and remove the biofilm to avoid, for example, strong odors or even health problems caused by these bacteria.

## 4. Conclusions

Overall, these results indicate that *P. fluorescens* and *P. aeruginosa* biofilms can be grown and analyzed on autoclaved woven cellulose substrates in a self-constructed biofilm growth chamber to analyze biofilm growth, and that standardized biofilm removal tests on fabrics can identify efficient biofilm removal formulations. Cellulose fibers have been shown to be an optimal scaffold for biofilm growth of *Pseudomonas* spp. It was proven via FE-SEM, 3D laser scanning microscopy, and epifluorescence microscopy that the biofilm can be successfully removed after 1 h of washing with a detergent- and enzyme-combining washing formulation. The combination of both the enzymes and detergent was significantly more efficient than either the enzyme- or detergent-based formulation alone. We also proved a significant reduction of viable *P. fluorescens* bacteria after treatment with the formulation composed of both detergent and enzymes. Although fixed *P. fluorescens* biofilms analyzed upon treatment for removal in real time via QCM measurements were less efficiently removed from the gold-coated quartz sensors compared to living biofilms on fabrics, a clear mass dissipation change upon washing with F1/2 could be monitored.

This clearly shows the potential of a combinatory approach including different active compounds targeting various antibiofilm strategies to make biofilm removal on textiles more efficient.

## Figures and Tables

**Table 1:** Parameters resulting from viscoelastic modeling of the overtone dependence of frequency and dissipation changes measured at the end of Stages 2 to 6 in the experiment shown in Figure 2.

Stage	d/nm	G′/kPa	G″/kPa	G′/G″	η′/mPa·s
2	20.2	60	237	0.25	1.26
3	17.8	76	215	0.35	1.14
4	20.8	68	214	0.32	1.13
5	15.0	85	213	0.40	1.13
6	10.9	94	211	0.45	1.12

All values are referenced to the baseline in hard water at the beginning of the experiment (i.e., Stage 1). d: thickness of the formed adlayer, G′: storage modulus, G″: loss modulus, η′: viscosity. Calculations were performed using the acoustic monolayer formalism (AMF, Fresnel-type) implemented in the QTM software developed by D. Johannsmann (2008) [61].

## Data Availability

The data presented in this study are available on request from the corresponding author.

## Supplementary Materials

The following supporting information can be downloaded at https://www.mdpi.com/article/10.3390/microorganisms11040892/s1, Figure S1: SEM images of *P. fluorescens* biofilms on cellulose substrates after washing with F1/2, Figure S2: FE-SEM images of *P. fluorescens* on cellulose substrates after washing with F1/2; Figure S3: Time-dependent changes in frequency and dissipation (corresponding to QCM measurements; Figure S4: SEM and optical microscopy images of fixed *P. fluorescens* biofilms on QCM sensors.

## References

[1] Chen H., Fink G.R. (2006). Feedback Control of Morphogenesis in Fungi by Aromatic Alcohols. Genes Dev..

[2] Litsky W. (1990). Wanted: Plastics with Antimicrobial Properties. Am. J. Public Health.

[3] Teufel L., Redl B. (2006). Improved Methods for the Investigation of the Interaction between Textiles and Micoorganisms. Lenzinger Berichte.

[4] Vishwakarma V. (2020). Impact of Environmental Biofilms: Industrial Components and Its Remediation. J. Basic Microbiol..

[5] Flemming H.C., Wingender J. (2010). The Biofilm Matrix. Nat. Rev. Microbiol..

[6] Sutherland I.W. (2001). The Biofilm Matrix—An Immobilized but Dynamic Microbial Environment. Trends Microbiol..

[7] Stewart P.S., Costerton J.W. (2001). Antibiotic Resistance of Bacteria in Biofilms. Lancet.

[8] Elasri M.O., Miller R.V. (1999). Study of the Response of a Biofilm Bacterial Community to UV Radiation. Appl. Environ. Microbiol..

[9] Cochran W.L., Mcfeters G.A., Stewart P.S. (2000). Reduced Susceptibility of Thin *Pseudomonas aeruginosa* Biofilms to Hydrogen Peroxide and Monochloramine. J. Appl. Microbiol..

[10] Gilbert P., Allison D.G., McBain A.J. (2002). Biofilms in Vitro and in Vivo: Do Singular Mechanisms Imply Cross-Resistance?. J. Appl. Microbiol..

[11] Stewart P.S. (2002). Mechanisms of Antibiotic Resistance in Bacterial Biofilms. Int. J. Med. Microbiol..

[12] Kelleher S.M., Habimana O., Lawler J., O’reilly B., Daniels S., Casey E., Cowley A. (2016). Cicada Wing Surface Topography: An Investigation into the Bactericidal Properties of Nanostructural Features. ACS Appl. Mater. Interfaces.

[13] Varshney S., Sain A., Gupta D., Sharma S. (2021). Factors Affecting Bacterial Adhesion on Selected Textile Fibres. Indian J. Microbiol..

[14] Oh J.K., Yegin Y., Yang F., Zhang M., Li J., Huang S., Verkhoturov S.V., Schweikert E.A., Perez-Lewis K., Scholar E.A. (2018). The Influence of Surface Chemistry on the Kinetics and Thermodynamics of Bacterial Adhesion. Sci. Rep..

[15] Fijan S., Turk S.Š. (2012). Hospital Textiles, Are They a Possible Vehicle for Healthcare-Associated Infections?. Int. J. Environ. Res. Public Health.

[16] Barlow C.G., Bloomfield S.F. (1982). An Investigation of Microbial Contamination in the Home. Epidemiol. Infect..

[17] Moccia G., Motta O., Pironti C., Proto A., Capunzo M., de Caro F. (2020). An Alternative Approach for the Decontamination of Hospital Settings. J. Infect. Public Health.

[18] Vonberg R.P., Weitzel-Kage D., Behnke M., Gastmeier P. (2011). Worldwide Outbreak Database: The Largest Collection of Nosocomial Outbreaks. Infection.

[19] Fazzeli H., Akbari R., Moghim S., Narimani T., Arabestani M.R., Ghoddousi A.R. (2012). Pseudomonas Aeruginosa Infections in Patients, Hospital Means, and Personnel’s Specimens. J. Res. Med. Sci..

[20] Silby M.W., Winstanley C., Godfrey S.A.C., Levy S.B., Jackson R.W. (2011). Pseudomonas Genomes: Diverse and Adaptable. FEMS Microbiol. Rev..

[21] Habash M.B., Park A.J., Vis E.C., Harris R.J., Khursigara C.M. (2014). Synergy of Silver Nanoparticles and Aztreonam against Pseudomonas Aeruginosa PAO1 Biofilms. Antimicrob. Agents Chemother..

[22] Gellatly S.L., Hancock R.E.W. (2013). Pseudomonas Aeruginosa: New Insights into Pathogenesis and Host Defenses. Pathog. Dis..

[23] Dheilly A., Soum-Soutéra E., Klein G.L., Bazire A., Compère C., Haras D., Dufour A. (2010). Antibiofilm Activity of the Marine Bacterium *Pseudoalteromonas* sp. Strain 3J6. Appl. Environ. Microbiol..

[24] Stewart P.S. (2003). Diffusion in Biofilms. J. Bacteriol..

[25] Gulati R., Sharma S., Sharma R.K. (2022). Antimicrobial Textile: Recent Developments and Functional Perspective. Polym. Bull..

[26] Karim N., Afroj S., Lloyd K., Oaten L.C., Andreeva D.V., Carr C., Farmery A.D., Kim I.D., Novoselov K.S. (2020). Sustainable Personal Protective Clothing for Healthcare Applications: A Review. ACS Nano.

[27] Simoncic B., Tomsic B. (2010). Structures of Novel Antimicrobial Agents for Textiles—A Review. Text. Res. J..

[28] Gupta A.K., Foley K.A. (2018). Evidence for Biofilms in Onychomycosis. G. Ital. Di Dermatol. Venereol..

[29] Periolatto M., Ferrero F., Vineis C., Varesano A., Gozzelino G. (2017). Novel Antimicrobial Agents and Processes for Textile Applications. Antibacterial Agents.

[30] Bridier A., Sanchez-Vizuete P., Guilbaud M., Piard J.C., Naïtali M., Briandet R. (2015). Biofilm-Associated Persistence of Food-Borne Pathogens. Food Microbiol..

[31] Lequette Y., Boels G., Clarisse M., Faille C. (2010). Using Enzymes to Remove Biofilms of Bacterial Isolates Sampled in the Food-Industry. Biofouling.

[32] Augustin M., Ali-Vehmas T., Atroshi F. (2004). Assessment of Enzymatic Cleaning Agents and Disinfectants against Bacterial Biofilms. J. Pharm. Sci..

[33] De Bivar Xavier J., Picioreanu C., van Loosdrecht M.C.M. (2005). A General Description of Detachment for Multidimensional Modelling of Biofilms. Biotechnol. Bioeng..

[34] Azeredo J., Azevedo N.F., Briandet R., Cerca N., Coenye T., Costa A.R., Desvaux M., Di Bonaventura G., Hébraud M., Jaglic Z. (2017). Critical Review on Biofilm Methods. Crit. Rev. Microbiol..

[35] Bajpai V., Dey A., Ghosh S., Bajpai S., Jha M.K. (2011). Quantification of Bacterial Adherence on Different Textile Fabrics. Int. Biodeterior. Biodegrad..

[36] Cattò C., Cappitelli F. (2019). Testing Anti-Biofilm Polymeric Surfaces: Where to Start?. Int. J. Mol. Sci..

[37] Buzalewicz I., Ulatowska-Jarża A., Gąsior-Głogowska M., Wolf-Baca M., Żyłka P. (2023). New Measurements Modalities for Multi-Parametric, Label-Free and Non-Contact Detection of Biofilm Formation on Stainless Steel and Glass Surfaces. Measurement.

[38] Gieroba B., Krysa M., Wojtowicz K., Wiater A., Pleszczyńska M., Tomczyk M., Sroka-Bartnicka A. (2020). The FT-IR and Raman Spectroscopies as Tools for Biofilm Characterization Created by Cariogenic Streptococci. Int. J. Mol. Sci..

[39] Nguyen U.T., Burrows L.L. (2014). DNase I and Proteinase K Impair Listeria Monocytogenes Biofilm Formation and Induce Dispersal of Pre-Existing Biofilms. Int. J. Food Microbiol..

[40] Walton C.L., Khalid M., Bible A.N., Kertesz V., Retterer S.T., Morrell-Falvey J., Cahill J.F. (2022). In Situ Detection of Amino Acids from Bacterial Biofilms and Plant Root Exudates by Liquid Microjunction Surface-Sampling Probe Mass Spectrometry. J. Am. Soc. Mass Spectrom..

[41] Neu T.R., Lawrence J.R. (2014). Advanced Techniques for in Situ Analysis of the Biofilm Matrix (Structure, Composition, Dynamics) by Means of Laser Scanning Microscopy. Methods Mol. Biol..

[42] Neu T.R., Lawrence J.R. (2014). Investigation of Microbial Biofilm Structure by Laser Scanning Microscopy. Adv. Biochem. Eng. Biotechnol..

[43] Bridier A., Dubois-Brissonnet F., Boubetra A., Thomas V., Briandet R. (2010). The Biofilm Architecture of Sixty Opportunistic Pathogens Deciphered Using a High Throughput CLSM Method. J. Microbiol. Methods.

[44] Deore P., Wanigasuriya I., Tsang Min Ching S.J., Brumley D.R., Van Oppen M.J.H., Blackall L.L., Hinde E. (2022). Fluorescence Lifetime Imaging Microscopy (FLIM): A Non-Traditional Approach to Study Host-Microbial Symbioses. Microbiol. Aust..

[45] Sugimoto S., Okuda K.I., Miyakawa R., Sato M., Arita-Morioka K.I., Chiba A., Yamanaka K., Ogura T., Mizunoe Y., Sato C. (2016). Imaging of Bacterial Multicellular Behaviour in Biofilms in Liquid by Atmospheric Scanning Electron Microscopy. Sci. Rep..

[46] Chatterjee S., Biswas N., Datta A., Dey R., Maiti P. (2014). Atomic Force Microscopy in Biofilm Study. Microscopy.

[47] Huang Q., Wu H., Cai P., Fein J.B., Chen W. (2015). Atomic Force Microscopy Measurements of Bacterial Adhesion and Biofilm Formation onto Clay-Sized Particles. Sci. Rep..

[48] Salazar J., Amer M.-À., Turó A., Castro N., Navarro M., Soto S., Gabasa Y., López Y., Chávez J.-A. (2023). Real-Time Detection of the Bacterial Biofilm Formation Stages Using QCM-Based Sensors. Chemosensors.

[49] O’Toole G.A. (2011). Microtiter Dish Biofilm Formation Assay. J. Vis. Exp..

[50] Salah Omer A., El Naeem G.A., Abd-Elhamid A.I., Farahat O.O.M., El-Bardan A.A., Soliman H.M.A., Nayl A.A. (2022). Adsorption of Crystal Violet and Methylene Blue Dyes Using a Cellulose-Based Adsorbent from Sugercane Bagasse: Characterization, Kinetic and Isotherm Studies. J. Mater. Res. Technol..

[51] Tran P.L., Hammond A.A., Mosley T., Cortez J., Gray T., Colmer-Hamood J.A., Shashtri M., Spallholz J.E., Hamood A.N., Reid T.W. (2009). Organoselenium Coating on Cellulose Inhibits the Formation of Biofilms by Pseudomonas Aeruginosa and Staphylococcus Aureus. Appl. Environ. Microbiol..

[52] Rajkowska K., Otlewska A., Guiamet P.S., Wrzosek H., Machnowski W. (2019). Pre-Columbian Archeological Textiles: A Source of Pseudomonas Aeruginosa with Virulence Attributes. Appl. Sci..

[53] Agustín M.R., Tarifa M.C., Vela-Gurovic M.S., Brugnoni L.I. (2023). Application of Natamycin and Farnesol as Bioprotection Agents to Inhibit Biofilm Formation of Yeasts and Foodborne Bacterial Pathogens in Apple Juice Processing Lines. Food Microbiol..

[54] Lindsay D., Von Holy A. (1997). Evaluation of Dislodging Methods for Laboratory-Grown Bacterial Biofilms. Food Microbiol..

[55] Emam H.E. (2019). Antimicrobial Cellulosic Textiles Based on Organic Compounds. 3 Biotech.

[56] Takashima M., Shirai F., Sageshima M., Ikeda N., Okamoto Y., Dohi Y. (2004). Distinctive Bacteria-Binding Property of Cloth Materials. Am. J. Infect. Control.

[57] Schmidt-Emrich S., Stiefel P., Rupper P., Katzenmeier H., Amberg C., Maniura-Weber K., Ren Q. (2016). Rapid Assay to Assess Bacterial Adhesion on Textiles. Materials.

[58] Tarifa M.C., Lozano J.E., Brugnoni L.I. (2018). Disinfection Efficacy over Yeast Biofilms of Juice Processing Industries. Food Research International.

[59] Johannsmann D. (2015). The Quartz Crystal Microbalance in Soft Matter Research.

[60] Sauerbrey G. (1959). Günter Verwendung von Schwingquarzen Zur Wägung Dünner Schichten Und Zur Mikrowägung. Z. Phys..

[61] Johannsmann D. (2008). Viscoelastic, Mechanical, and Dielectric Measurements on Complex Samples with the Quartz Crystal Microbalance. Phys. Chem. Chem. Phys..

[62] Molobela I., Cloete T.E., Beukes M. (2010). Protease and Amylase Enzymes for Biofilm Removal and Degradation of Extracellular Polymeric Substances (EPS) Produced by Pseudomonas Fluorescens Bacteria. Afr. J. Microbiol. Res..

