# Supplementary material for: Monitoring Growth and Removal of Pseudomonas Biofilms on Cellulose-Based Fabrics
Source: Microorganisms. 2023 Mar 30;11(4):892. doi: 10.3390/microorganisms11040892 (PMC10143030; doi:10.3390/microorganisms11040892)
Supplement: Supplementary file 1 [file microorganisms-11-00892-s001.zip › microorganisms-2281036-supplementary.pdf]

Supplementary Materials

Supplementary material S1.

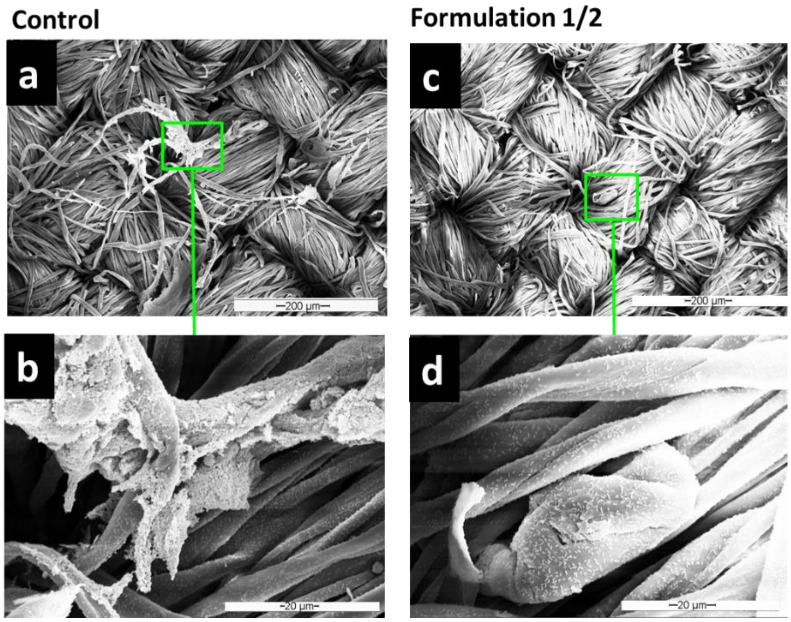

Figure S1. SEM images of *P. fluorescens* biofilms grown for 48 h on cellulose substrates after 1 h washing with formulation 1/2 (c, d) in comparison to non-treated controls (a, b). Lower (a, c) and higher (b, d) magnifications of the sections indicated in green are shown. (addition to Fig. 3., independently repeated experiment)

Supplementary material S2.

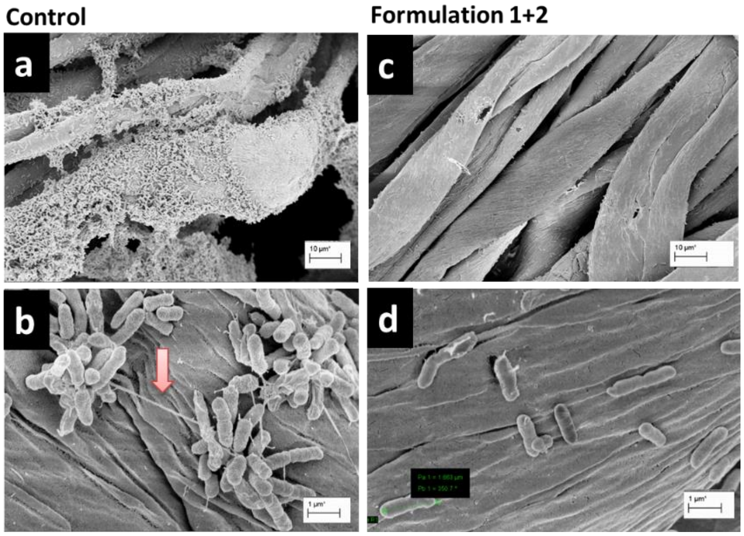

Figure S2. FE-SEM images of *P. fluorescens* biofilms grown for 48 h on cellulose substrates after 1 h washing with formulation 1/2 (c, d) in comparison to non-treated controls (a, b). Red arrow indicates inter-bacterial connections via pili. Lower (upper row) and higher (lower row) magnifications are shown. Length of a *P. fluorescens* bacterium is measures with 1,66 µm in d (as addition to Fig. 3. with higher resolution and repeated experiment in tilted plastic well plate)

Supplementary material S3.

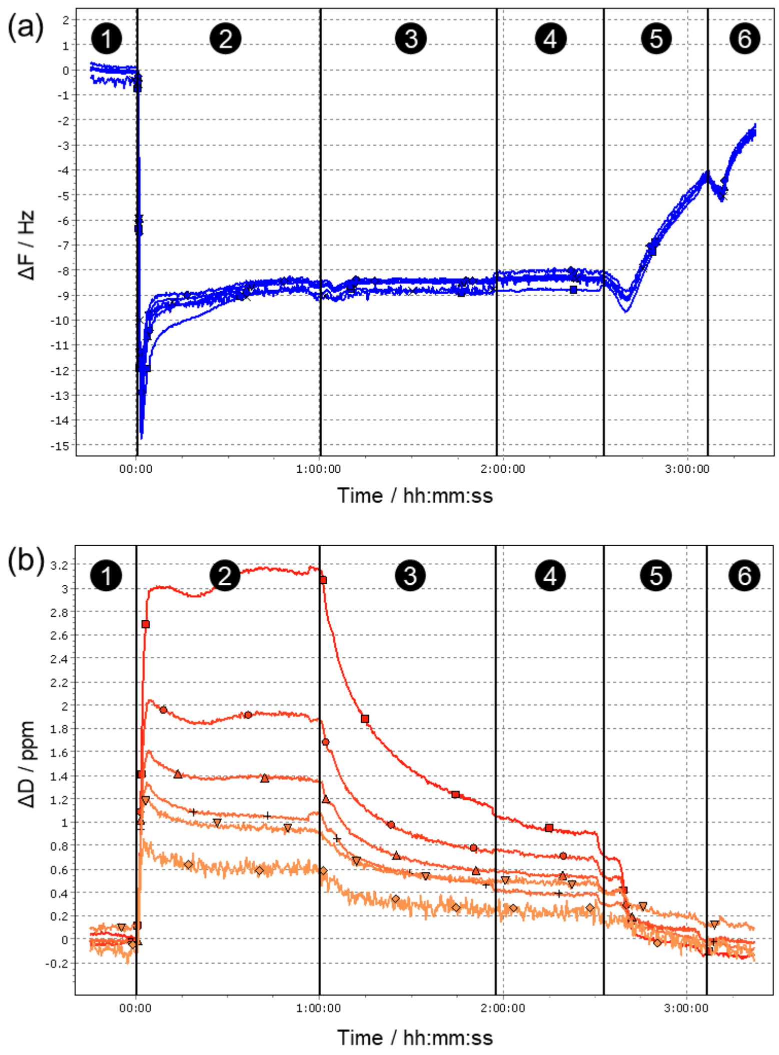

Figure S3. Time-dependent changes in (a) frequency and (b) dissipation during sequential incubation of a blank Au-coated surface with (1) hard water, (2) detergent in hard water (F1), (3) enzyme and detergent in hard water (F1/2), (4) detergent in hard water (F1), (5) hard water, and (6) deionized water. For both ΔF and ΔD, signals from different overtones (n) of the main resonance are shown (squares: n = 3, circles: n = 5, triangles: n = 7, inverted triangles: n = 9, diamonds: n = 11, crosses: n = 13).

Supplementary material S4.

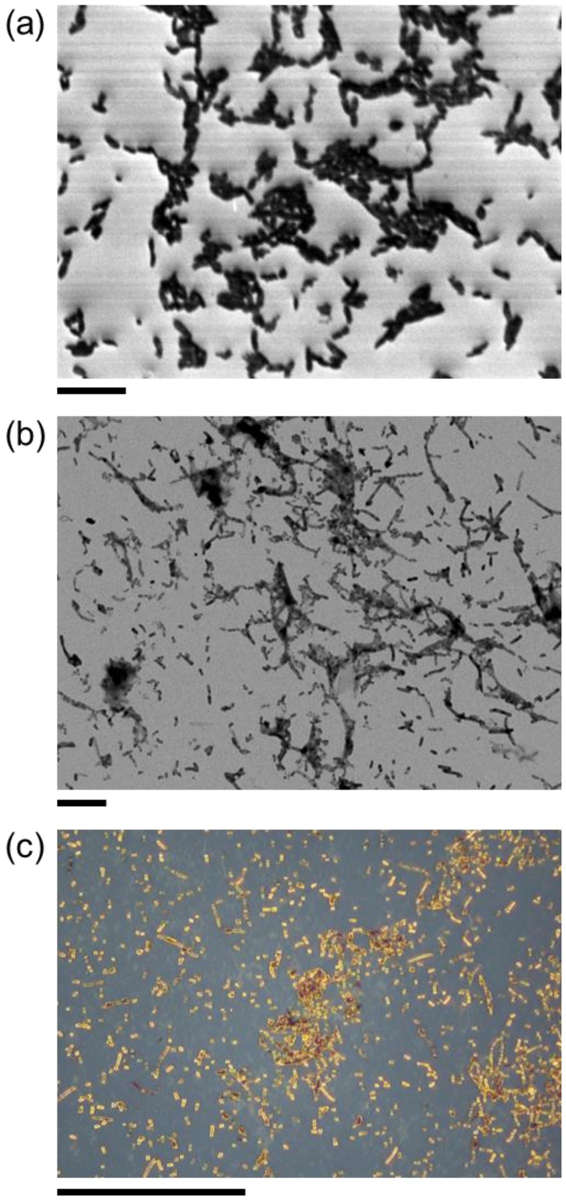

Figure S4. SEM (a-b) and optical microscopy (c) images of fixed *P. fluorescens* biofilm on QCM sensors (a) before and (b-c) after the QCM experiment shown in Figure 2, Shown is one exemplary picture out of 2 independent QCM measurements. Scale bars are 10 µm.
